# Self-reported health-related quality of life of the general population in Alberta, Canada during the COVID-19 pandemic

**DOI:** 10.1186/s41687-022-00518-y

**Published:** 2022-10-12

**Authors:** Jiabi Wen, Fatima Al Sayah, Roland Simon, Markus Lahtinen, Jeffrey A. Johnson, Arto Ohinmaa

**Affiliations:** 1 School of Public Health, 3-267 Edmonton Clinic Health Academy, University of Alberta, 11405 - 87 Ave NW, Edmonton, AB T6G 1C9 Canada; 2 Health Quality Council of Alberta, Calgary, AB Canada

**Keywords:** COVID-19, Health-related quality of life, EQ-5D-5L, General population, Alberta, Population norms

## Abstract

**Background:**

The COVID-19 pandemic has impacted various aspects of people’s life and wellbeing around the world. This study aimed to examine the impact of the COVID-19 pandemic on health-related quality of life (HRQL), measured by the EQ-5D-5L, amongst the general population in the province of Alberta, Canada, and explore whether the impact varied across population subgroups based on age, gender, and dwelling.

**Methods:**

Data came from two waves of a repeated cross-sectional population-based survey, the *COVID-19 Experiences and Impact Survey*, administered by the Health Quality Council of Alberta. The first data collection (survey 1: n = 8790) was during May/June 2020 and the second (survey 2: n = 9263) during Oct 2020. We examined the comparability of weighted survey data and their representativeness to Alberta’s general population. We then explored between-survey differences in EQ-5D-5L index, EQ-VAS and dimension responses, and differences across subgroups within each survey. We compared HRQL of the pooled sample (survey 1&2) with the Alberta population norms data from the pre-pandemic period.

**Results:**

Mean EQ-5D-5L index and EQ-VAS scores were 0.81 (0.15) and 72.54 (18.57), and 0.82 (0.14) and 71.98 (18.96) in surveys 1 and 2, respectively. The anxiety/depression dimension had the most reported problems (survey 1: 69.5%, survey 2: 70.2%). Respondents aged 16–24 or 75 and older, who identified themselves as a woman, or residing in urban areas had significantly lower EQ-5D-5L index scores compared to their counterparts in both surveys. Between-survey differences were not substantially different. Comparing the pooled sample with the pre-pandemic Alberta population norms, EQ-5D-5L index scores (0.82 vs. 0.84) and EQ-VAS scores (72.26 vs. 77.40) were significantly lower, and respondents aged 16–44, women, or urban residents were more impacted. More problems were reported in the anxiety/depression (69.9% vs. 37.2%) and usual activities dimensions (40.5% vs. 26.0%) during the pandemic period, especially for respondents aged 16–44, women, and those residing in urban areas.

**Conclusions:**

Lower HRQL was reported during the COVID-19 pandemic compared to pre-pandemic HRQL in this population, with anxiety/depression and usual activities affected the most. People who were younger, women, and residing in urban areas were most impacted. The government responses to COVID-19 policies during population outbreaks should consider the needs of Albertans in these particular groups.

**Supplementary Information:**

The online version contains supplementary material available at 10.1186/s41687-022-00518-y.

## Background

The COVID-19 pandemic has both directly and indirectly impacted population health worldwide. As of May 2022, the cumulative number of COVID-19 confirmed cases was 520 million worldwide, with 3.83 million in Canada [1]. Patients infected with COVID-19 have various symptoms and multiple organs being impacted [2, 3]. Some patients may have post-acute COVID-19 syndrome which lasts more than 4 weeks or even chronically [4]. With massive medical resources devoted to treating COVID-19 and concerns about protecting patients from infection, people without COVID-19 may experience delayed healthcare [5], which potentially threatens people’s health. In addition, several systematic reviews have shown increasing prevalence of a variety of mental health problems during the pandemic, e.g., anxiety [6], depression [7], posttraumatic stress and psychological stress [8]. Statistics Canada reported that Canadians had worse self-perceived mental health in 2020 compared to pre-pandemic, especially among females [9]. Varied factors contributed to these mental illnesses, e.g., COVID-19 infection [10] or fear of infection [11], social and economic factors such as restrictions on social gatherings and travelling [12], and unemployment [13].

Emerging evidence suggests that general population’s health-related quality of life (HRQL) was impacted during the pandemic. Using tools like the EQ-5D-5L [14], research in China [15], Morocco [16], Portugal [17], the US [18], and Vietnam [19] has identified lower self-reported HRQL among the general population during the pandemic compared to pre-pandemic reference values (population norms). Before the pandemic, young people had the highest HRQL in many jurisdictions [20, 21], but they tended to experience greater HRQL loss during the pandemic [15, 18]. It remains unknown how the COVID-19 pandemic has impacted population HRQL in Alberta or Canada. However, in a study focusing on emergency department and primary care settings in Alberta, Canada, HRQL measured by the EQ-5D-5L among patients seeking primary care had a remarkable decline, especially for females and young people [22].

The future trajectory of COVID-19 is anticipated to be endemic and perhaps with seasonal peaks [23], so the impact of COVID-19 on HRQL is likely to exist at least for several years and potentially it will re-shape population norms. Some subpopulations, e.g., younger people, might be emerging vulnerable populations during the pandemic. Exploring HRQL of the general population and possibly identifying new population norms help governments and society better understand population health and invoke their attention on related health issues and those vulnerable populations. Besides, population norms are useful guides to interpret HRQL scores observed in individuals, groups of patients, or subpopulations [24], which further informs resource allocation and decision-making. Updated population norms as a valid reference value for comparisons are warranted. In Alberta, the use of EQ-5D-5L to measure HRQL is popular, and pre-COVID EQ-5D-5L population norms [25] were available and applied in many clinical studies. Exploring new population norms will be relevant and informative to end-users.

During the COVID-19 pandemic in Canada, there has been a lack of information on its impact on HRQL among the population. Considering the potential existence of new population norms and the associated benefits for policy-making, we aimed to examine the impact of the pandemic on HRQL, measured by the EQ-5D-5L, among the general population in the Province of Alberta.

## Methods

### Data

The data came from two waves of a repeated cross-sectional population-based survey conducted by the Health Quality Council of Alberta (HQCA), the *COVID-19 Experiences and Impact Survey*, which asked respondents questions concerning public health measures, health system access, and the support are available to stay informed, well, and protected during the pandemic [26]. This online survey invited over 15,000 Albertans to participate via several email lists and was also promoted via social media, news, online advertisement, and the HQCA website [27]. The first wave of data collection (survey 1) was between May 25 and June 29, 2020, and the second (survey 2) took place between October 1–31, 2020. HRQL was measured using the EQ-5D-5L. Questions on demographics, socioeconomic status, underlying health conditions, health behaviors, and the COVID-19 situation were included in the survey. The study procedures were approved by the Health Research Ethics Board at the University of Alberta (Pro00101660).

### Pandemic and public health response during the survey period

The 2020 Alberta population was 4.4 million individuals, with 81% living in an urban area. The first confirmed COVID-19 case in Alberta was reported on March 5, 2020, and the first wave peaked on April 30, 2020, with 3022 active cases. The first wave continued until September 2020, with 18,175 cases in total. The second wave started in October 2020, and by October 19, there were 3,138 active cases, which was the highest reported in Alberta since March 2020 [28]. The second wave ended in February 2021 with a total of 115,550 positive tested cases in the province [29].

The government of Alberta initiated public health responses in March 2020, with restrictions on all gatherings, travel, and non-essential business, and the closure of schools and universities. Stage 1 of re-opening in mid-May 2020 lifted some restrictions, and with stage 2 in June 2020 more restrictions were lifted. Stage 2 continued until November 2020 when restrictions were re-instituted. The HQCA survey 1 began towards the end of stage 1 and continued into stage 2, and the survey 2 occurred near the end of stage 2.

### Measures

The EQ-5D-5L is a generic preference-based HRQL measure, which includes a descriptive system and a visual analogue scale (EQ-VAS) [30]. The descriptive system has five dimensions: mobility, self-care, usual activities, pain/discomfort, and anxiety/depression, each with five levels: 1 = no problems, 2 = slight problems, 3 = moderate problems, 4 = severe problems, 5 = extreme problems, describing 3125 distinct health states. The EQ-VAS is a vertical visual analogue scale ranging from 0 “worst health you can imagine” to 100 “best health you can imagine”, and is used to assess overall respondent health [30]. The EQ-5D-5L is recommended to measure HRQL in Alberta and Canada [31]. The Canadian EQ-5D-5L value set is available to calculate EQ-5D-5L preference-based index scores, ranging from − 0. 149 to 0.949, with higher scores indicating better HRQL [32]. EQ-5D-5L population norms were published for the Alberta population in 2018, with a mean index of 0.84 and a mean VAS score of 77.4 [25]. Alberta population norms also reported dimensional proportions and HRQL distribution for age group, sex, and residence within Alberta Health Service’s five geographical zones.

The HQCA survey collected information on age group, gender, and residential location. We re-grouped the age variable based on age group categories of the Alberta norms population. For the gender variable, we only analyzed HRQL among those who identified themselves as a man or a woman. Postcodes, which could identify the residence in health zones, had many missing values. A potential substitute variable is dwelling (urban/rural) as health zone-specific HRQL in Alberta population norms showed urban health zones (Calgary and Edmonton) had slightly better HRQL compared to the rural zones (North, Central, and South zones) [25]. However, this variable was only administered in the second survey.

Other variables we used were for examining the comparability and representatives of our sample, and these included socio-demographics (levels of education, whether born in Canada, ethnicity, work sector, household income, and financial situation), underlying health conditions (diabetes, heart/vascular disease, liver/kidney disease, having or had cancer, lung disease, and autoimmune disease), health behaviors (smoking and cannabis consumption), and the COVID-19 status (had been infected or not).

### Data analysis

All analyses were performed in Stata version 14. We weighted the survey sample based on age and gender within each health zone to increase the representativeness. We did not include other socio-demographic variables for weighting factors, since more variables would limit the number of observations in each stratum. For respondents with missing health zone, we treated them as a group. Our robustness analyses showed the HRQL outcomes were insensitive to our weighting strategy, when comparing with no weighting implemented and weighting on a subsample excluding respondents having missing health zones (data not shown).

We examined the sample representativeness before and after weighting. Reference data for demographics and socioeconomic status variables, except for unemployment rate, were from the most recent 2016 Canadian census [33]; unemployment rates were from the Government of Alberta [34]; underlying health conditions were from Alberta Health’s Interactive Health Data Application [35]; health behaviors were from the 2020 Canadian Community Health Survey [36] and National Cannabis Survey [37]; and COVID-19 infection data were from the Government of Alberta [29].

We explored weighted HRQL in survey 1 and survey 2 in the whole population and then in terms of age group, gender, and dwelling to see if there are any between-wave differences. EQ-5D-5L index scores and EQ-VAS were estimated with the mean and standard deviation (SD). To analyze dimensional responses, we examined the weighted proportion of respondents having no problems (level 1) versus any problems (levels 2, 3, 4 and 5), having no problems (level 1) versus having mild-moderate problems (levels 2–3) and severe-extreme problems (levels 4–5), and having problems in each level. We used a Wald test for EQ-5D-5L index scores and EQ-VAS and a design-based F-test for dimensional responses to identify between-wave differences in HRQL. If no differences were observed, we would pool the survey 1 and survey 2 data to represent HRQL during the pandemic and then compared it with the pre-pandemic Alberta population norms. For EQ-5D-5L index scores and EQ-VAS, we used the survey mean command followed by a Lincom (linear combination of estimators) command, and for dimensional responses, we used the survey proportion command followed by a Lincom command. We used the minimally important difference (MID) to examine the magnitude of the differences in the EQ-5D-5L index (MID = 0.037) [38] and in EQ-VAS (MID = 7.0). For dimension responses, we deemed the difference was a small magnitude if the absolute difference in proportions was within 5%.

## Results

### Sample

Observations were excluded (Survey 1: 21.49%, Survey 2: 21.43%) if data were missing for age (Survey 1: 13.15%; Survey 2: 13.37%), gender (Survey 1: 13.87%; Survey 2: 14.22%), EQ-5D-5L (Survey 1: 15.43%; Survey 2: 13.93%), or EQ-VAS (Survey 1: 15.97%; Survey 2: 17.06%). The total sample included 18,053 observations, with 8790 in survey 1 and 9263 in survey 2. Excluded respondents were a little older, had slightly more women, were a little less urban and lower educated, were more from a non-white ethnicity, had lower household income, had a poorer financial situation, and had lower HRQL compared to the included sample (Table 1).

**Table 1:** Data management and missing

	Overall	Survey 1	Survey 2
Original sample	22,985	11,196	11,789
Final sample (included)	18,053	8790	9263
Missing in age, gender, EQ-5D-5L, EQ-VAS (%) (excluded)	4932 (21.46)	2406 (21.49)	2526 (21.43)
Missing in age (%)	3048 (13.26)	1472 (13.15)	1576 (13.37)
Missing in gender (%)	3229 (14.05)	1553 (13.87)	1676 (14.22)
Missing in EQ-5D-5L (%)	3369 (14.66)	1727 (15.43)	1642 (13.93)
Missing in EQ-VAS (%)	3799 (16.53)	1788 (15.97)	2011 (17.06)

Comparison of included/excluded respondents (survey 1 and survey 2 combined)		
	Included (%)	Excluded (%)
Age group	16–24: 455 (2.52)	30 (1.75)
	25–44: 6411 (35.51)	588 (34.27)
	45–64: 7596 (42.08)	723 (42.13)
	65–74: 2833 (15.69)	281 (16.38)
	75+: 758 (4.20)	94 (5.48)
Gender	Man: 4652 (25.77)	366 (21.49)
	Woman: 13,401 (74.23)	1337 (78.51)
Urban/rural (survey 2 only)	Rural: 1544 (19.26)	202 (23.03)
	Urban: 6474 (80.74)	675 (76.97)
Education	Grade school or some high school: 314 (1.75)	65 (3.66)
	Completed high school 1729 (9.63)	228 (12.83)
	Postsecondary certificate, diploma or degree: 15,906 (88.62)	1484 (83.51)
Born in Canada	Yes: 16,036 (89.14)	1624 (88.36)
	No: 1954 (10.86)	214 (11.64)
Ethnicity	White: 13,888 (87.63)	1193 (82.96)
	Non-white: 2040 (12.37)	245 (17.04)
Working sector	Retired: 3534 (20.88)	311 (20.23)
	Unemployed: 997 (5.89)	95 (6.18)
	Student: 305 (1.80)	20 (1.30)
	Agriculture: 341 (2.01)	45 (2.93)
	Education: 1670 (9.87)	159 (10.34)
	Healthcare: 2628 (15.53)	240 (15.61)
	Social services: 486 (2.87)	39 (2.54)
	Service/hospitality: 694 (4.10)	81 (5.27)
	Construction/manufacturing: 764 (4.51)	59 (3.84)
	Industry/engineering/technology: 1556 (9.19)	109 (7.09)
	Other: 3952 (23.35)	379 (24.66)
Household income	< $25,000: 1016 (6.26)	143 (9.67)
	$25,000–$50,000: 2151 (13.25)	231 (15.62)
	$50,000–$100,000: 5485 (33.78)	498 (33.67)
	$100,000–$150,000: 3619 (22.29)	295 (19.95)
	> $150,000: 3965 (24.42)	312 (21.10)
Financial situation	Very comfortable: 1644 (9.29)	130 (7.57)
	Comfortable: 5494 (31.04)	441 (25.67)
	Modestly comfortable: 5597 (31.63)	513 (29.86)
	Tight: 3,167 (17.89)	373 (21.71)
	Very tight or poor: 1796 (10.15)	261 (15.19)
EQ-5D-5L	0.81 (0.15)	0.772 (0.17)
EQ-VAS	72.67 (18.42)	72.25 (19.38)

Survey 1 and survey 2 respondents had similar distributions across age groups, gender, place of birth, and health behaviors (smoking and cannabis consumption). Respondents in survey 1 were more likely to have higher education, to be of non-white ethnicities, to be workers in healthcare and social services sectors, to have higher household income and a comfortable financial situation, and to have higher prevalence of some underlying health conditions (diabetes, heart/vascular disease, having or had cancer, liver/kidney disease, lung disease, and autoimmune disease) compared to those in wave 2. Additionally, fewer respondents in survey 1 reported being students, working in agriculture or construction/manufacturing sectors, and experiencing COVID-19 infection (Additional file 1: Table S1).

Implementing sample weights increased the sample representativeness. Compared to the general Alberta population, respondents in both surveys were more educated, more likely to identify as “white”, more likely to be born in Canada, and had higher rates of self-reported cannabis consumption. Survey respondents reported lower unemployment levels and smoking rates. Additionally, more survey respondents were at both the upper and lower end of the household income distribution compared to the 2016 census [33]. Although there were variations in reporting chronic diseases that made direct comparison difficult, overall rates were similar between the survey and the Alberta population values. Population characteristics measured by the remaining variables were overall similar to the general population values (Additional file 1: Table S1).

### HRQL in the overall population

**Fig. 1:**
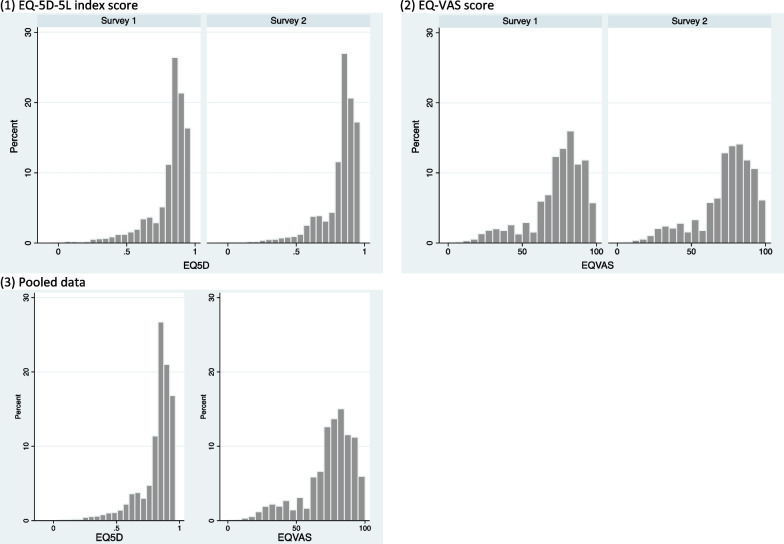
Histograms of EQ-5D-5L index scores and EQ-VAS scores in survey 1, survey 2, and the pooled dataset

**Fig. 2:**
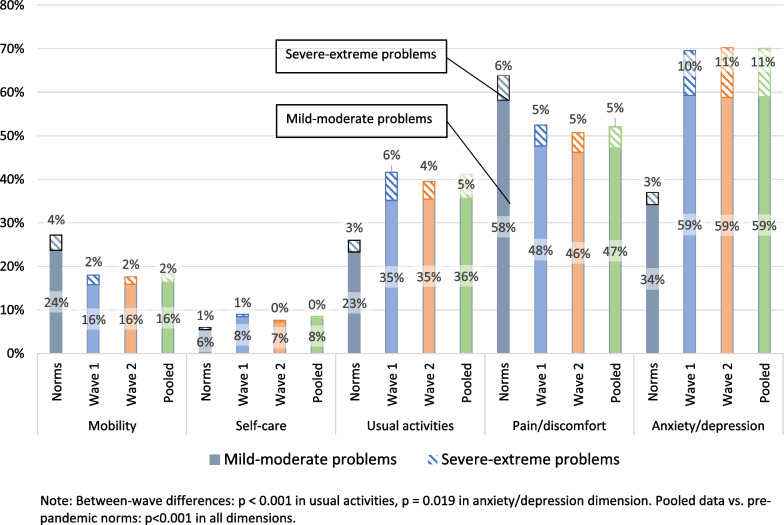
Proportion of respondents reporting problems in each EQ-5D-5L dimension (the Alberta norm, survey 1, survey 2, and combined). *Note*: Between-wave differences: *p* < 0.001 in usual activities, *p* = 0.019 in anxiety/depression dimension. Pooled data versus pre-pandemic norms: *p* < 0.001 in all dimensions

The mean EQ-5D-5L index scores were 0.81 (0.15) and 0.82 (0.14) and mean EQ-VAS scores were 72.54 (18.57) and 71.98 (18.96) in surveys 1 and 2, respectively. Histograms of EQ-5D-5L index scores and EQ-VAS in survey 1 and survey 2 showed skewed distribution (Fig. 1). The median (interquartile range) for EQ-5D-5L index score was 0.87 (0.78, 0.91) in both surveys, and the median (interquartile range) for EQ-VAS was 76 (65, 85) in the survey 1 and 75 (65, 85) in the survey 2. The ceiling effect (respondents reporting “no problems” in all dimensions) was 6.3% and 6.7% for survey 1 and survey 2 samples, respectively. There were no significant differences in the EQ-5D-5L index score or the EQ-VAS score between the two surveys (Table 2). The dimension with the most reported problems was anxiety/depression (69.5% vs. 70.2% for survey 1 and survey 2), followed by the pain/discomfort dimension (52.5% vs. 50.7% for survey 1 and survey 2) and usual activities dimension (41.6% vs. 39.5% for survey 1 and survey 2). The dimension with the least reported problems was self-care (9.0% vs. 7.6% for survey 1 and survey 2) (Fig. 2). Dimensional between-survey differences were only significant in the usual activities dimension (*p* = 0.001) and anxiety/depression dimension (*p* = 0.019), but the magnitudes of differences were small (Additional file 1: Table S2).

**Table 2:** Weighted EQ-5D-5L index and EQ-VAS scores in either survey

	EQ-5D-5L index scores					EQ-VAS scores				
	Survey 1 (Sample N = 8790)		Survey 2 (Sample N = 9263)		Survey 1 versus Survey 2	Survey 1 (Sample N = 8790)		Survey 2 (Sample N = 9263)		Survey 1 versus Survey 2
	Mean (s.d.)	Comparison across groups	Mean (s.d.)	Comparison across groups		Mean (s.d.)	Comparison across groups	Mean (s.d.)	Comparison across groups	
Total	0.81 (0.15)		0.82 (0.14)		*p* = 0.127	72.54 (18.57)		71.98 (18.96)		*p* = 0.239
*Age group*										
16–24	0.78 (0.18)	Versus 25–44: *p* = 0.001 Versus 45–64: *p* = 0.005 Versus 65–74: *p* = 0.032 Versus 75+: *p* = 0.761	0.80 (0.12)	Versus 25–44: *p* = 0.018 Versus 45–64: *p* = 0.022 Versus 65–74: *p* = 0.143 Versus 75+: *p* = 0.536	*p* = 0.188	69.87 (21.07)	Versus 25–44: *p* = 0.086 Versus 45–64: *p* = 0.048 Versus 65–74: *p* = 0.007 Versus 75+: *p* = 0.091	67.79 (19.51)	Versus 25–44: *p* = 0.017 Versus 45–64: *p* = 0.003 Versus 65–74: *p* < 0.001 Versus 75+: *p* = 0.001	*p* = 0.369
25–44	0.83 (0.14)	Versus 45–64: *p* = 0.077 Versus 65–74: *p* = 0.003 Versus 75+: *p* < 0.001	0.82 (0.14)	Versus 45–64: *p* = 0.802 Versus 65–74: *p* = 0.103 Versus 75+: *p* = 0.003	*p* = 0.644	72.66 (17.86)	Versus 45–64: *p* = 0.521 Versus 65–74: *p* = 0.016 Versus 75+: *p* = 0.797	71.94 (18.87)	Versus 45–64: *p* = 0.070 Versus 65–74: *p* = 0.002 Versus 75+: *p* = 0.015	*p* = 0.243
45–64	0.82 (0.15)	Versus 65–74: *p* = 0.134 Versus 75+: *p* < 0.001	0.82 (0.15)	Versus 65–74: *p* = 0.128 Versus 75+: *p* = 0.003	*p* = 0.201	73.05 (18.37)	Versus 65–74: *p* = 0.050 Versus 75+: *p* = 0.903	72.91 (18.88)	Versus 65–74: *p* = 0.82 Versus 75+: *p* = 0.104	*p* = 0.788
65–74	0.81 (0.16)	Versus 75+: *p* < 0.001	0.82 (0.14)	Versus 75+: *p* = 0.031	*p* = 0.313	74.37 (18.70)	Versus 75+: *p* = 0.178	74.00 (18.06)	Versus 75+: *p* = 0.554	*p* = 0.637
75+	0.77 (0.18)		0.79 (0.16)		*p* = 0.173	72.92 (19.18)		74.70 (16.11)		*p* = 0.204
*Gender*										
Woman	0.80 (0.16)	Versus man: *p* < 0.001	0.80 (0.15)	Versus man: *p* < 0.001	*p* = 0.216	72.20 (18.67)	Versus man: *p* = 0.300	71.88 (18.37)	Versus man: *p* = 0.762	*p* = 0.498
Man	0.83 (0.14)		0.83 (0.14)		*p* = 0.280	72.86 (17.84)		72.08 (20.10)		*p* = 0.336
*Urban/rural residence*^1^										
Urban			0.81 (0.15)	Versus rural: *p* = 0.015				71.32 (18.92)	Versus rural: *p* = 0.002	
Rural			0.83 (0.14)					73.79 (18.95)		

^1^Information on urban/rural residences was only collected in survey 2. Urban/rural residence was a substitute variable for health zones in the Alberta population norms. Residing in urban corresponds to the Calgary/Edmonton health zone, and residing in rural areas corresponds to the North, Central, and South health zones

### HRQL in population subgroups

Some subgroups had significantly lower EQ-5D-5L index scores than their counterparts in both surveys: respondents aged 16–24 or aged 75 and older versus respondents aged 25–74, respondents who identified as women versus those who identified as men, and respondents residing in urban areas versus those residing in rural areas. In terms of EQ-VAS, respondents aged 16–24 and residing in urban areas had lower HRQL compared with their counterparts, but there were no statistically significant differences for EQ-VAS across gender subgroups (Table 2).

All subgroups except for respondents aged 65 and older had more problems reported in usual activities, pain/discomfort, and anxiety/depression dimensions and were less impacted by mobility and self-care dimensions. For respondents aged 65 and older, there were also numerous problems in the mobility dimension. Younger respondents, those who self-identified as a woman, and reside in urban areas reported higher proportions of problems in the usual activities and anxiety/depression dimensions, compared to their counterparts. Older respondents, and those who self-identified as a woman reported more problems in the pain/discomfort dimension compared to their counterparts (Additional file 1: Fig SA1–SA9).

There were no between-survey differences in EQ-5D-5L and EQ-VAS scores in each subgroup (Table 2). All age and gender subgroups had some dimensions with significant between-survey differences, but the magnitude of those differences was small (Additional file 1: Fig SA1–SA7).

### HRQL in the population during and pre-pandemic

Given a few between-survey differences in HRQL, we estimated HRQL in the pooled dataset to reflect HRQL during the pandemic (Fig. 2, Table 3). Compared with the pre-pandemic Alberta norms, HRQL during the pandemic was lower for both the EQ-5D-5L index score (0.84 vs. 0.82, *p* < 0.001) and EQ-VAS score (77.40 vs. 72.26, *p* < 0.001), respectively. However, both differences were within the MID range. EQ-5D-5L and EQ-VAS scores in subgroups defined by age, gender, and dwelling were all lower than the pre-pandemic reference value. Most of these differences were significant, and such differences were greater than the MID for respondents aged 16–44, women and respondents residing in urban areas (Table 3).

**Table 3:** Weighted EQ-5D-5L index and EQ-VAS and comparison with Alberta norms

	EQ-5D-5L index (weighted)			EQ-VAS (weighted)		
	Pooled	Norms	Pooled versus norms	Pooled	Norms	Pooled VERSUS NOrms
	(Sample N = 18,053)			(Sample N = 18,053)		
Total	0.82 (0.15)	0.84 (0.14)	Diff: 0.02 *p* < 0.001	72.26 (18.77)	77.4 (17.1)	Diff: 5.14 *p* < 0.001
*Age group*						
16–24	0.79 (0.06)	0.88 (0.10)	Diff: **0.09**^1^ *p* < 0.001	68.78 (8.26)	81.6 (14.2)	Diff: **12.82** *p* < 0.001
25–44	0.83 (0.13)	0.87 (0.11)	Diff: **0.04** *p* < 0.001	72.30 (17.75)	79.8 (15.1)	Diff: **7.50** *p* < 0.001
45–64	0.82 (0.18)	0.83 (0.16)	Diff: 0.01 *p* < 0.001	72.97 (21.78)	76.3 (17.9)	Diff: 3.33 *p* < 0.001
65–74	0.81 (0.19)	0.82 (0.15)	Diff: 0.01 *p* = 0.033	74.18 (23.53)	75.5 (18.1)	Diff: 1.32 *p* < 0.001
75+	0.78 (0.14)	0.80 (0.15)	Diff: 0.02 *p* = 0.020	73.87 (14.23)	73.9 (18.0)	Diff: 0.03 *p* = 0.965
*Gender*						
Woman	0.80 (0.19)	0.85 (0.14)	Diff: **0.05** *p* < 0.001	72.03 (22.83)	78.3 (16.1)	Diff: 6.27 *p* < 0.001
Man	0.83 (0.10)	0.86 (0.13)	Diff: 0.03 *p* < 0.001	72.47 (13.50)	77.3 (16.1)	Diff: 4.83 *p* < 0.001
*Urban/rural residence*^2^						
Urban	0.81 (0.15)	0.85^**3**^ (0.13)	Diff: **0.04**^**3**^ *p* < 0.001	71.32 (18.92)	78.5 (16.0)	Diff: **7.18** *p* < 0.001
Rural	0.83 (0.14)	0.84^**2**^ (0.15)	Diff: 0.01 *p* = 0.026	73.79 (18.95)	76.9 (17.6)	Diff: 3.11 *p* < 0.001

^1^Bold indicating the difference was greater than the minimally important difference

^2^Information on urban/rural residence was only collected in survey 2. Urban/rural residence was a substitute variable for health zones in the Alberta population norms. Residing in urban corresponds to the Calgary/Edmonton health zone, and residing in rural areas corresponds to the North, Central, and South health zones

^3^The table shows the Calgary health zone reference value. Mean EQ5D index (s.d.) and EQ-VAS score (s.d.) in the Edmonton health zone were 0.85 (0.14) and 77.6 (16.7). EQ-5D-5L index (Diff: 0.04, *p* < 0.001) and EQ-VAS score (Diff: 6.28, *p* < 0,001) during the pandemic were significantly different from the Edmonton reference value

People aged 16–24 had the highest HRQL in the pre-pandemic norms and HRQL decreased with increasing age, however, mean EQ-5D-5L and EQ-VAS scores for respondents aged 16–24 in the pooled dataset were even lower than those for respondents aged 65–74. Respondents residing in urban areas had higher HRQL in the pre-pandemic reference value than those residing in rural areas, and the relation is vice versa during the pandemic (Table 3).

Responses to all EQ-5D-5L dimensions during the pandemic were significantly different from pre-pandemic norms, and the differences in usual activities, pain/discomfort, and anxiety/depression dimensions were of a greater magnitude. During the pandemic, respondents reported fewer problems in the pain/discomfort dimension (64.0% vs. 51.6%) and more problems in the usual activities (26.0% vs. 40.5%) and anxiety/depression dimensions (37.2% vs. 69.9%). Especially in the anxiety/depression dimension, a lot more severe (level 4) to extreme (level 5) problems were reported (2.8% vs. 10.9%) (Fig. 2 and Additional file 1: Table S2). All subgroups reported fewer problems in the pain/discomfort dimension, especially for respondents aged 45–64 (71.4% vs. 57.1%) and self-identified as a man (62.3% vs. 47.4%). All subgroups except for respondents aged 65–74 had more problems in the usual activities dimension, especially for respondents aged 16–44 (16–24: 13.3% vs. 52.1%; 25–44: 15.6% vs. 40.5%) and residing in urban areas (23.8% (Calgary) or 23.9% (Edmonton) vs. 41.3%). All subgroups reported a significantly higher proportion of problems in the anxiety/depression dimension, especially for respondents aged 16–44 (16–24: 44.0% vs. 83.1%; 25–44: 39.1% vs. 75.8%), self-identified as a woman (39.8% vs. 75.2%), and residing in urban areas (36.7% (Calgary) or 39.9% (Edmonton) vs. 72.5%). (Additional file 1: Fig A1–A9 and Additional file 1: Tables S3, S4).

## Discussion

This study showed that HRQL of the Alberta general population, as measured by the EQ-5D-5L, was lower during the COVID-19 pandemic as compared to the pre-pandemic population norms, with a significantly higher proportion of problems in the usual activities and anxiety/depression dimensions and a lower proportion of problems in the pain/discomfort dimension. This finding is robust in the overall population and across subgroups, which suggests the existence of a shifting of population norms during the pandemic.

The finding that more problems were reported in the anxiety/depression dimension during the pandemic is consistent with previous literature in other populations. In EQ-5D-5L studies from China [15], Morocco [16], Portugal [17], the US [18], and Vietnam [19], the anxiety/depression dimension had the highest proportion of problems reported. In our study, we also found the differences in anxiety/depression dimension between pandemic and pre-pandemic were greater among respondents aged 16–44, women, and those residing in urban areas. According to a September 2021 report from Statistics Canada, there was a decline in perceived mental health, especially among females, compared to pre-pandemic [9]. We further identified that respondents aged 16–24 and women suffered more in the anxiety/depression dimension. This is consistent with a systematic review on COVID-19 and mental health, which showed that females and adults aged 40 years old or younger had more risks for mental distress [39]. Potential reasons for younger people and women facing more mental health problems can be that they may be less resilient and may have less emotion regulation and coping strategies [40, 41], they are more distressed about school and work responsibilities [42], and experience uncertain conditions and financial stress [43, 44].

Usual activities is another dimension notably impacted, especially for younger groups of the population, which is similar to findings from the Chinese [15] study. Usual activities of the EQ-5D-5L refers to work, study, housework, and family or leisure activities, and the higher level of problems reported in this dimension might be explained by restrictions related to COVID-19. During the survey 1 and survey 2 periods, there were restrictions from governments and institutions related to the above activities, e.g., lockdowns, restrictions on non-essential business, and school closure. Population aged 16–44 were more impacted in this dimension when compared to the norms. People in this age group were more likely to be university students or have a child attending pre-school/daycare and K-12 schools. Existing literature has shown dramatic changes in academic and social life for students in higher education during the pandemic [45] and work-life conflicts for workers with young children at home [46]. These factors put them at greater risk of experiencing problems in terms of usual activities people compared to their counterparts [47, 48].

We observed a decreased prevalence of problems in the pain/discomfort dimension during the pandemic, which ran contrary to other studies. Many believed reducing physical activities, prolonged homestay and increased sitting time, teleworking, and e-learning would increase the risk of pain and discomfort [49, 50]. There might be several reasons that we did not observe such an effect. First, our sample might have a lower proportion of people with underlying health conditions, compared to the population participating in the study on Alberta population norms. Second, the two surveys were administered early in the pandemic, and the long-term effect on pain/discomfort may not have emerged at that point.

Respondents who aged younger, were women, or resided in an urban area tended to be more impacted during the pandemic. They were more impacted in the anxiety/depression and usual activities dimensions, as compared to population norms. This leads to lower HRQL measured by the EQ-5D-5L. Similar age and gender effects on HRQL were previously reported [15, 18, 22]. Little is known about the relationship between the dwelling and HRQL. In some jurisdictions, rural areas seemed to have more COVID-19 crises [51]. In Alberta and likely other jurisdictions with similar contexts, residents in urban areas had stricter restrictions on behaviour. Thus, there would be greater impacts on everyday life, including higher chances of infection because of more COVID-19 cases. Indeed, they showed lower HRQL compared with those living in rural areas. For these subgroups substantially impacted by the COVID-19 pandemic, it is important that policies and restrictions take into account their needs.

This study supports the existing evidence suggesting lower HRQL among the general population during the COVID-19 pandemic. Given the fact that HRQL measured by the EQ-5D-5L during the pandemic was dramatically different from the pre-pandemic norms, clinical studies conducted during the pandemic may be advised to use more applicable reference population norm values, such as those reported in this study. Although the survey only collected HRQL in the early waves of the pandemic, when COVID-19 was relatively well-controlled in Alberta, restrictions during this time were moderate compared to the rest of the pandemic. Since restrictions varied during the pandemic, HRQL collected in this survey might reflect an average level of HRQL during the pandemic. Since the beginning of 2022, many jurisdictions have been taking steps to return to normal (pre-pandemic state), but COVID-19 will still likely impact in the following months or years, with the possible emergence of other serious variants and more strict restrictions required. Population norms during the pandemic would be relevant for the next several years, and more research is needed to explore HRQL in the later waves of the pandemic.

The main limitation of this study was that the original sample was not reflective of the Alberta population as a whole. We applied sample weights to increase the comparability and representativeness. If respondents were assigned a relatively greater weight but their HRQL was not a reflection of their demographic group, this would lead to over-representing issues. Second, we collected self-reported gender identity in the survey, while the pre-pandemic norms showed HRQL by sex. Since we did not observe gender difference in HRQL, the comparison between HRQL during the pandemic and pre-pandemic is still likely valid. Third, we used the dwelling variable (urban/rural residential location) which was administered only in survey 2 as a proxy for urban/rural health zones. We were unable to observe the HRQL in terms of dwelling in survey 1, and therefore fewer observations to estimate HRQL by dwelling. Besides, the rural health zones have cities that can be categorized as urban residential areas. However, although the dwelling variable did not well represent the health zone, we still observed lower HRQL among those who reside in urban areas.

## Conclusion

This study showed that HRQL was lower and the distributional characteristics were different during the COVID-19 pandemic, as compared to pre-COVID-19 population norms, with anxiety/depression and usual activities dimensions mostly affected, and younger people, women, and people residing in urban areas suffering the most. To cope with the disproportionate impacts, policy-makers should consider the health risks and HQRL problems for specific subgroups and their health and social needs.

## Supplementary Information

**Additional file 1.** Supplementary Tables 1-4 and Supplementary Figures A1-A9.

## Data Availability

The data that support the findings of this study are available from the Health Quality Council of Alberta but restrictions apply to the availability of these data. Data are available from the authors upon reasonable request and with permission of the Health Quality Council of Alberta.

## Abbreviations

HRQL: Health-related quality of life
HQCA: Health Quality Council of Alberta

## References

[1] WHO Coronavirus (COVID-19) Dashboard (2021) Retrieved November 30, 2021, from https://covid19.who.int

[2] Kakodkar P, Kaka N, Baig M (n.d.) A comprehensive literature review on the clinical presentation, and management of the pandemic coronavirus disease 2019 (COVID-19). Cureus 12(4):e7560. 10.7759/cureus.7560 10.7759/cureus.7560 PMC7138423 32269893

[3] Deshmukh V, Motwani R, Kumar A, Kumari C, Raza K (2021). Histopathological observations in COVID-19: a systematic review. J Clin Pathol.

[4] Nalbandian A, Sehgal K, Gupta A, Madhavan MV, McGroder C, Stevens JS, Cook JR, Nordvig AS, Shalev D, Sehrawat TS, Ahluwalia N, Bikdeli B, Dietz D, Der-Nigoghossian C, Liyanage-Don N, Rosner GF, Bernstein EJ, Mohan S, Beckley AA, Seres DS, Choueiri TK, Uriel N, Ausiello JC, Accili D, Freedberg DE, Baldwin M, Schwartz A, Brodie D, Garcia CK, Elkind MSV, Connors JM, Bilezikian JP, Landry DW, Wan EY (2021). Post-acute COVID-19 syndrome. Nat Med.

[5] Rosenbaum L (2020). The untold toll—the pandemic’s effects on patients without Covid-19. N Engl J Med.

[6] Santabárbara J, Lasheras I, Lipnicki DM, Bueno-Notivol J, Pérez-Moreno M, López-Antón R, De la Cámara C, Lobo A, Gracia-García P (2021). Prevalence of anxiety in the COVID-19 pandemic: an updated meta-analysis of community-based studies. Prog Neuro-Psychopharmacol Biol Psychiatry.

[7] Bueno-Notivol J, Gracia-García P, Olaya B, Lasheras I, López-Antón R, Santabárbara J (2021). Prevalence of depression during the COVID-19 outbreak: a meta-analysis of community-based studies. Int J Clin Health Psychol.

[8] Cooke JE, Eirich R, Racine N, Madigan S (2020). Prevalence of posttraumatic and general psychological stress during COVID-19: a rapid review and meta-analysis. Psychiatry Res.

[9] Government of Canada SC (2021) Self-perceived mental health and mental health care needs during the COVID-19 pandemic. Retrieved September 15, 2021, from https://www150.statcan.gc.ca/n1/pub/45-28-0001/2021001/article/00031-eng.htm

[10] Aiyegbusi OL, Hughes SE, Turner G, Rivera SC, McMullan C, Chandan JS, Haroon S, Price G, Davies EH, Nirantharakumar K, Sapey E, Calvert MJ (2021). Symptoms, complications and management of long COVID: a review. J R Soc Med.

[11] Şimşir Z, Koç H, Seki T, Griffiths MD (2021). The relationship between fear of COVID-19 and mental health problems: a meta-analysis. Death Stud.

[12] Pfefferbaum B, North CS (2020). Mental health and the Covid-19 pandemic. N Engl J Med.

[13] Kawohl W, Nordt C (2020). COVID-19, unemployment, and suicide. Lancet Psychiatry.

[14] Herdman M, Gudex C, Lloyd A, Janssen Mf, Kind P, Parkin D, Bonsel G, Badia X (2011). Development and preliminary testing of the new five-level version of EQ-5D (EQ-5D-5L). Qual Life Res.

[15] Mao Z, Chen B, Wang W, Kind P, Wang P (2021). Investigating the self-reported health status of domestic and overseas Chinese populations during the COVID-19 pandemic. Int J Environ Res Public Health.

[16] Azizi A, Achak D, Aboudi K, Saad E, Nejjari C, Nouira Y, Hilali A, Youlyouz-Marfak I, Marfak A (2020). Health-related quality of life and behavior-related lifestyle changes due to the COVID-19 home confinement: dataset from a Moroccan sample. Data Brief.

[17] Ferreira LN, Pereira LN, da Fé Brás M, Ilchuk K (2021). Quality of life under the COVID-19 quarantine. Qual Life Res.

[18] Hay JW, Gong CL, Jiao X, Zawadzki NK, Zawadzki RS, Pickard AS, Xie F, Crawford SA, Gu NY (2021). A US population health survey on the impact of COVID-19 using the EQ-5D-5L. J Gen Intern Med.

[19] Tran BX, Nguyen HT, Le HT, Latkin CA, Pham HQ, Vu LG, Le XTT, Nguyen TT, Pham QT, Ta NTK, Nguyen QT, Ho CSH, Ho RCM (2020). Impact of COVID-19 on economic well-being and quality of life of the Vietnamese during the national social distancing. Front Psychol.

[20] Jiang R, Janssen MFB, Pickard AS (2021). US population norms for the EQ-5D-5L and comparison of norms from face-to-face and online samples. Qual Life Res.

[21] Yang Z, Busschbach J, Liu G, Luo N (2018). EQ-5D-5L norms for the urban Chinese population in China. Health Qual Life Outcomes.

[22] Al Sayah F, Lahtinen M, Simon R, Higgins B, Ohinmaa A, Johnson JA (2022). The impact of COVID-19 pandemic on health-related quality of life of adults visiting emergency departments and primary care settings in Alberta. Can J Public Health.

[23] Telenti A, Arvin A, Corey L, Corti D, Diamond MS, García-Sastre A, Garry RF, Holmes EC, Pang PS, Virgin HW (2021). After the pandemic: perspectives on the future trajectory of COVID-19. Nature.

[24] Fayers PM, Machin D (2007) Clinical interpretation. In: Quality of life: the assessment, analysis and interpretation of patient-reported outcomes. Wiley, pp 427–455. 10.1002/9780470024522.ch18

[25] Alberta PROMs & EQ-5D Research & Support Unit (APERSU) (2018) Alberta population norms for EQ-5D-5L. School of Public Health, University of Alberta, Edmonton, Canada. Retrieved from https://apersu.ca/wp-content/uploads/2021/02/Alberta-Norms-Report_APERSU.pdf

[26] COVID-19 Experiences and Impact Survey (2020) HQCA. Retrieved from https://hqca.ca/covid-19/

[27] Health Quality Council of Alberta (2020) COVID-19 Experiences and Impact Survey, voices of Albertans, May–Jun 2020. Retrieved from https://www.hqca.ca/wp-content/uploads/2020/09/COVID-Report-09_16.pdf

[28] CBC News (2020) Alberta hits new pandemic peak for active COVID-19 cases|CBC News. CBC. Retrieved from https://www.cbc.ca/news/canada/edmonton/alberta-covid-19-coronavirus-active-cases-pandemic-1.5768458

[29] Government of Alberta (2020) COVID-19 Alberta statistics. Retrieved from https://www.alberta.ca/stats/covid-19-alberta-statistics.htm#total-cases

[30] Devlin NJ, Brooks R (2017). EQ-5D and the EuroQol Group: past, present and future. Appl Health Econ Health Policy.

[31] Canadian Agency for Drugs & Technologies in Health (CADTH) (2017) Guidelines for the economic evaluation of health technologies: Canada, 4th ed. CADTH, Ottawa. Retrieved from https://www.cadth.ca/about-cadth/how-we-do-it/methods-and-guidelines/guidelines-for-the-economic-evaluation-of-health-technologies-canada

[32] Xie F, Pullenayegum E, Gaebel K, Bansback N, Bryan S, Ohinmaa A, Poissant L, Johnson JA (2016). A time trade-off-derived value set of the EQ-5D-5L for Canada. Med Care.

[33] Statistics Canada (2017) Census profile. 2016 Census. Statistics Canada Catalogue no. 98-316-X2016001. Retrieved from https://www12.statcan.gc.ca/census-recensement/2016/dp-pd/prof/index.cfm?Lang=E

[34] Government of Alberta (2020) Unemployment rate. Economic dashboard. Retrieved from https://economicdashboard.alberta.ca/unemployment

[35] Government of Alberta (n.d.) Interactive health data application. Retrieved from http://www.ahw.gov.ab.ca/IHDA_Retrieval/selectSubCategory.do

[36] Statistics Canada (2021) Table 13-10-0096-01 health characteristics, annual estimates. 10.25318/1310009601-eng

[37] Statistics Canada (2021) Table 13-10-0383-01 prevalence of cannabis use in the past three months, self-reported. 10.25318/1310038301-eng

[38] McClure NS, Sayah FA, Xie F, Luo N, Johnson JA (2017). Instrument-defined estimates of the minimally important difference for EQ-5D-5L index scores. Value Health J Int Soc Pharmacoecon Outcomes Res.

[39] Xiong J, Lipsitz O, Nasri F, Lui LMW, Gill H, Phan L, Chen-Li D, Iacobucci M, Ho R, Majeed A, McIntyre RS (2020). Impact of COVID-19 pandemic on mental health in the general population: a systematic review. J Affect Disord.

[40] Barber SJ, Kim H (2021). COVID-19 worries and behavior changes in older and younger men and women. J Gerontol Ser B.

[41] Varma P, Junge M, Meaklim H, Jackson ML (2021). Younger people are more vulnerable to stress, anxiety and depression during COVID-19 pandemic: a global cross-sectional survey. Prog Neuropsychopharmacol Biol Psychiatry.

[42] Liu CH, Zhang E, Wong GTF, Hyun S, Hahm HC (2020). Factors associated with depression, anxiety, and PTSD symptomatology during the COVID-19 pandemic: clinical implications for US young adult mental health. Psychiatry Res.

[43] Pieh C, Budimir S, Probst T (2020). The effect of age, gender, income, work, and physical activity on mental health during coronavirus disease (COVID-19) lockdown in Austria. J Psychosom Res.

[44] Ochnik D, Rogowska AM, Kuśnierz C, Jakubiak M, Schütz A, Held MJ, Arzenšek A, Benatov J, Berger R, Korchagina EV, Pavlova I, Blažková I, Aslan I, Çınar O, Cuero-Acosta YA (2021). Mental health prevalence and predictors among university students in nine countries during the COVID-19 pandemic: a cross-national study. Sci Rep.

[45] Aristovnik A, Keržič D, Ravšelj D, Tomaževič N, Umek L (2020). Impacts of the COVID-19 pandemic on life of higher education students: a global perspective. Sustainability.

[46] Schieman S, Badawy PJ, Milkie MA, Bierman A (2021). Work-life conflict during the COVID-19 pandemic. Socius.

[47] Churchill B (2021). COVID-19 and the immediate impact on young people and employment in Australia: a gendered analysis. Gend Work Organ.

[48] Long D, Haagsma JA, Janssen MF, Yfantopoulos JN, Lubetkin EI, Bonsel GJ (2021). Health-related quality of life and mental well-being of healthy and diseased persons in 8 countries: Does stringency of government response against early COVID-19 matter?. SSM Popul Health.

[49] Caputo EL, Reichert FF (2020). Studies of physical activity and COVID-19 during the pandemic: a scoping review. J Phys Act Health.

[50] Buomprisco G, Ricci S, Perri R, Sio SD (2021). Health and telework: new challenges after COVID-19 pandemic. Eur J Environ Public Health.

[51] Ranscombe P (2020). Rural areas at risk during COVID-19 pandemic. Lancet Infect Dis.

