# Supplementary material for: Self-reported health-related quality of life of the general population in Alberta, Canada during the COVID-19 pandemic
Source: J Patient Rep Outcomes. 2022 Oct 12;6:109. doi: 10.1186/s41687-022-00518-y (PMC9556143; doi:10.1186/s41687-022-00518-y)
Supplement: Supplementary file 1 — Additional file 1. Supplementary Tables 1-4 and Supplementary Figures A1-A9. [file 41687_2022_518_MOESM1_ESM.docx]

**Supplementary material (Wen J, et al. JPRO 2022)**

In this document, all notations are numbered using superscripts (e.g., ^1, 2, 3,^ …), and all citations are numbered with square brackets (e.g., [1], [2], [3], …).

Supplementary Table 1: General characteristics of respondents, by survey and pooled, and their comparisons to the Alberta population

|  | Unweighted data | | | Weighted data | | | Alberta population (%) | Survey 1 vs. Survey 2 | |
| --- | --- | --- | --- | --- | --- | --- | --- | --- | --- |
| Characteristics | Survey 1  (N = 8790) | Survey  (N= 9263) | Pooled  (N = 18053) | Survey 1  % | Survey 2  % | Overall  % |  | Unweighted | Weighted |
| Age group |  |  |  |  |  |  |  |  |  |
| 16-24 | 263 (2.71) | 237 (2.34) | 455 (2.52) | 14.88 | 15.51 | 15.20 | 13.97 [1] | p < 0.001 | p = 0.534 |
| 25-44 | 3423 (35.54) | 3661 (35.49) | 6411 (35.51) | 38.99 | 37.06 | 38.00 | 37.91 [1] |  |  |
| 45-64 | 3989 (40.88) | 4391 (43.21) | 7596 (42.08) | 30.52 | 31.03 | 30.78 | 32.63 [1] |  |  |
| 65-74 | 1569 (16.09) | 1551 (15.32) | 2833 (15.69) | 9.42 | 9.71 | 9.57 | 9.04 [1] |  |  |
| 75+ | 480 (4.79) | 373 (3.64) | 758 (4.20) | 6.2 | 6.68 | 6.44 | 6.40 [1] |  |  |
| Gender |  |  |  |  |  |  |  |  |  |
| Woman | 6741 (76.69) | 6660 (71.90) | 13401 (74.23) | 48.46 | 49.27 | 48.88 | 50.51 [1] | p < 0.001 | p = 0.507 |
| Man | 2049 (23.31) | 2603 (28.10) | 4652 (25.77) | 51.54 | 50.73 | 51.12 | 49.49 [1] |  |  |
| Urban/rural residence |  |  |  |  |  |  |  |  |  |
| Urban |  | 1544 (19.26) | 1544 (19.26) |  | 18.68 | 18.68 | 16.4 [2] |  |  |
| Rural |  | 6474 (80.74) | 6474 (80.74) |  | 81.32 | 81.32 | 83.6 [2] |  |  |
| Education |  |  |  |  |  |  |  |  |  |
| Grade school or some high school | 138 (1.58) | 176 (1.91) | 314 (1.75) | 3.04 | 3.73 | 3.39 | 16.86 [1] | p < 0.001 | p = 0.069 |
| Completed high school | 740 (8.47) | 989 (10.73) | 1729 (9.63) | 10.36 | 12.56 | 11.49 | 27.94 [1] |  |  |
| Postsecondary certificate, diploma  or degree | 7857 (89.95) | 8049 (87.36) | 15906 (88.62) | 86.60 | 83.71 | 85.12 | 55.19 [1] |  |  |
| Born in Canada |  |  |  |  |  |  |  |  |  |
| Yes | 7744 (88.45) | 8292 (89.79) | 16036 (89.14) | 88.77 | 89.43 | 89.11 | 76.99 [1] | p = 0.004 | p = 0.343 |
| No | 1011 (11.55) | 943 (10.21) | 1954 (10.86) | 11.23 | 10.57 | 10.89 | 23.01 [1] |  |  |
| Ethnicity |  |  |  |  |  |  |  |  |  |
| Arab | 46 (0.61) | 25 (0.30) | 71 (0.45) | 0.78 | 0.38 | 0.58 | 1.4 [1] | p < 0.001 | p = 0.022 |
| Black | 71 (0.94) | 31 (0.38) | 102 (0.64) | 1.22 | 0.35 | 0.77 | 3.3 [1] |  |  |
| East Asian | 113 (1.49) | 92 (1.11) | 205 (1.29) | 2.05 | 2.03 | 2.04 | 4.8 [1] |  |  |
| Indigenous | 232 (3.06) | 252 (3.05) | 484 (3.05) | 3.01 | 3.07 | 3.04 | 7.64 [1] |  |  |
| Latin American | 80 (1.05) | 73 (0.88) | 153 (0.97) | 1.48 | 1.09 | 1.28 | 1.4 [1] |  |  |
| South Asian | 104 (1.37) | 67 (0.81) | 171 (1.08) | 1.84 | 1.50 | 1.66 | 5.8 [1] |  |  |
| Southeast Asian | 87 (1.15) | 70 (0.85) | 157 (0.99) | 1.72 | 1.67 | 1.69 | 5.3 [1] |  |  |
| White | 6592 (86.85) | 7296 (88.35) | 13888 (87.63) | 84.31 | 85.92 | 85.14 | 68.91 [1] |  |  |
| Other | 265 (3.49) | 352 (4.26) | 617 (3.89) | 3.60 | 3.99 | 3.80 |  |  |  |
| Working sector |  |  |  |  |  |  |  |  |  |
| Retired | 1810 (22.05) | 1724 (19.77) | 3534 (20.88) | 16.16 | 15.90 | 16.02 |  | p < 0.001 | p = 0.001 |
| Unemployed | 452 (5.51) ^3^ | 545 (6.25) ^3^ | 997 (5.89) ^3^ | 6.52 | 5.93 | 6.22 | May/June: 15.65, Oct: 10.8 ^2^ [3] |  |  |
| Student | 154 (1.88) | 151 (1.73) | 305 (1.90) | 6.54 | 7.71 | 7.14 |  |  |  |
| Agriculture | 101 (1.23) | 240 (2.75) | 341 (2.01) | 1.23 | 2.60 | 1.94 |  |  |  |
| Education | 844 (10.28) | 826 (9.47) | 1670 (9.87) | 8.11 | 7.37 | 7.73 |  |  |  |
| Healthcare | 1436 (17.50) | 1192 (13.67) | 2628 (15.23) | 13.89 | 11.31 | 12.57 |  |  |  |
| Social services | 273 (3.33) | 213 (2.44) | 486 (2.87) | 3.01 | 2.17 | 2.58 |  |  |  |
| Service/hospitality | 304 (3.70) | 390 (4.47) | 694 (4.10) | 4.77 | 5.04 | 4.91 |  |  |  |
| Construction/Manufacturing | 281 (3.42) | 483 (5.54) | 764 (4.51) | 5.55 | 6.90 | 6.25 |  |  |  |
| Industry/Engineering/Technology | 730 (8.89) | 826 (9.47) | 1556 (9.19) | 13.29 | 12.96 | 13.12 |  |  |  |
| Other | 1822 (22.20) | 2130 (24.43) | 3952 (23.35) | 20.93 | 22.11 | 21.54 |  |  |  |
| Household income, before tax |  |  |  |  |  |  |  |  |  |
| < $25,000 | 517 (6.56) | 499 (5.97) | 1016 (6.26) | 7.76 | 8.13 | 7.95 | 6.15 [1] | p = 0.004 | p = 0.011 |
| $25,000-$49,999 | 1067 (13.54) | 1084 (12.97) | 2151 (13.25) | 14.35 | 13.67 | 14.00 | 9.87 [1] |  |  |
| $50,000-$99,999 | 2600 (32.99) | 2885 (34.53) | 5485 (33.78) | 30.95 | 34.45 | 32.75 | 20.38 [1] |  |  |
| $100,000-$149,999 | 1698 (21.55) | 1921 (22.99) | 3619 (22.29) | 21.23 | 21.85 | 21.55 | 46.42 [1] |  |  |
| > $150,000 | 1998 (25.36) | 1967 (23.54) | 3965 (24.42) | 25.70 | 21.90 | 23.75 | 17.18 [1] |  |  |
| Financial situation |  |  |  |  |  |  |  |  |  |
| Very comfortable | 865 (10.05) | 779 (8.57) | 1644 (9.29) | 9.77 | 8.08 | 8.90 |  | p < 0.001 | p = 0.027 |
| Comfortable | 2788 (32.40) | 2706 (29.76) | 5494 (31.04) | 31.02 | 29.07 | 30.02 |  |  |  |
| Modestly comfortable | 2708 (31.47) | 2708 (31.47) | 5597 (31.63) | 30.32 | 31.19 | 30.77 |  |  |  |
| Tight | 1494 (17.36) | 1494 (17.36) | 3167 (17.89) | 18.99 | 20.09 | 19.56 |  |  |  |
| Very tight | 751 (8.73) | 751 (8.73) | 1796 (10.15) | 9.89 | 11.56 | 10.75 |  |  |  |
| Current situation on COVID-19 |  |  |  |  |  |  |  |  |  |
| Never infected | 8242 (93.96) | 8909 (96.52) | 17151 (95.27) | 94.03 | 95.98 | 95.03 |  | p < 0.001 | p < 0.001 |
| May have been infected | 507 (5.78) | 284 (3.08) | 791 (4.39) | 5.73 | 3.54 | 4.61 |  |  |  |
| Infected and recovered | 23 (0.26) | 37 (0.40) | 60 (0.33) | 0.24 | 0.48 | 0.36 | End of Oct: 0.65 ^3^ [4] |  |  |
| Underlying health conditions and health behaviors |  |  |  |  |  |  |  |  |  |
| Diabetes | 600 (6.83) | 537 (5.80) | 1137 (6.30) | 5.75 | 5.04 | 5.39 | 7.85 [5] | p = 0.004 | p = 0.088 |
| Heart disease or vascular disease | 635 (7.22) | 485 (5.24) | 1120 (6.20) | 6.56 | 4.90 | 5.71 | 6.5 ^4^ [5] | p < 0.001 | p = 0.004 |
| Liver or kidney disease | 161 (1.74) | 343 (1.90) | 182 (2.07) | 1.92 | 1.36 | 1.63 | 0.37 ^5^ [5] | p = 0.102 | p = 0.016 |
| Having or had cancer | 410 (4.66) | 345 (3.72) | 755 (4.18) | 3.71 | 3.07 | 3.38 |  | p = 0.002 | p = 0.035 |
| Lung disease | 1278 (14.54) | 1135 (12.25) | 2413 (13.37) | 13.47 | 11.26 | 12.34 | 15.12 ^6^ [5] | p < 0.001 | p = 0.005 |
| Autoimmune disease | 802 (9.12) | 723 (7.81) | 1525 (8.45) | 6.78 | 5.80 | 6.28 | 3.01 ^7^ [5] | p = 0.001 | p = 0.038 |
| Smoke | 488 (5.55) | 481 (5.19) | 969 (5.37) | 5.59 | 5.22 | 5.40 | 14.10 [6] | p = 0.285 | p = 0.643 |
| Cannabis | 1924 (21.97) | 1981 (21.55) | 3905 (21.76) | 28.04 | 26.20 | 27.1 | 21.70 [7] | p = 0.491 | p = 0.145 |
| Note:   1. Given that our study population is very large, when comparing survey 1 and survey 2 statistics, even though the two population had a slight difference, a significant p-value would still be observed. 2. The unemployment rate reference value only encountered employed and unemployed population, which is different from our survey structure. If ruling out the retired population in our survey sample, the new unemployment rate for survey 1 and survey 2 would be 7.07% and 7.79%, respectively. 3. This prevalence statistics was generated by total cases of COVID-19 in Alberta on June 30 and Oct 31 divided by the population. 4. This estimate was generated by grouping atrial fibrillation, congestive heart failure, and ischemic heart disease. 5. This estimate was generated by grouping liver cirrhosis and end-stage kidney disease in the database. 6. This estimate was generated by grouping asthma, bronchiectasis disease, and chronic obstructive pulmonary disease in the database. 7. This estimate was generated by grouping inflammatory bowel disease, multiple sclerosis, and rheumatoid arthritis in the database. | | | | | | | | | |

Supplementary Table 2: Weighted dimensional responses in survey 1 and 2, and comparison with the Alberta norms

|  |  | Survey 1  (Sample N =8790) | Survey 2  (Sample N = 9263) | Survey 1 vs. Survey 2 | Norm [8] | Pooled | Norm vs. Pooled |
| --- | --- | --- | --- | --- | --- | --- | --- |
| Mobility | Level 1 | 82.0% | 82.4% | p = 0.261 | 72.8% | 82.2% | p < 0.001 |
|  | Level 2 | 10.6% | 11.1% |  | 15.2% | 10.9% |  |
|  | Level 3 | 5.2% | 4.7% |  | 8.5% | 5.0% |  |
|  | Level 4 | 1.9% | 1.5% |  | 3.0% | 1.7% |  |
|  | Level 5 | 0.3% | 0.2% |  | 0.5% | 0.3% |  |
| Self-care | Level 1 | 91.0% | 92.4% | p = 0.121 | 94.1% | 91.7% | p < 0.001 |
|  | Level 2 | 6.5% | 5.8% |  | 3.7% | 6.1% |  |
|  | Level 3 | 2.0% | 1.4% |  | 1.8% | 1.7% |  |
|  | Level 4 | 0.3% | 0.2% |  | 0.3% | 0.3% |  |
|  | Level 5 | 0.2% | 0.1% |  | 0.2% | 0.1% |  |
| Usual activities | Level 1 | 58.4% | 60.5% | p = 0.001 | 74.0% | 59.5% | p < 0.001 |
|  | Level 2 | 22.6% | 22.1% |  | 15.3% | 22.3% |  |
|  | Level 3 | 12.6% | 13.3% |  | 8.0% | 13.0% |  |
|  | Level 4 | 3.8% | 2.8% |  | 1.8% | 3.3% |  |
|  | Level 5 | 2.6% | 1.3% |  | 0.9% | 1.9% |  |
| Pain/discomfort | Level 1 | 47.5% | 49.3% | p = 0.125 | 36.0% | 48.4% | p < 0.001 |
|  | Level 2 | 31.9% | 32.3% |  | 38.8% | 32.1% |  |
|  | Level 3 | 15.8% | 13.9% |  | 19.4% | 14.8% |  |
|  | Level 4 | 4.1% | 3.8% |  | 4.5% | 3.9% |  |
|  | Level 5 | 0.7% | 0.7% |  | 1.2% | 0.7% |  |
| Anxiety/depression | Level 1 | 30.5% | 29.8% | p = 0.019 | 62.8% | 30.1% | p < 0.001 |
|  | Level 2 | 36.6% | 34.2% |  | 23.4% | 35.4% |  |
|  | Level 3 | 22.7% | 24.6% |  | 10.8% | 23.7% |  |
|  | Level 4 | 6.0% | 8.1% |  | 1.9% | 7.1% |  |
|  | Level 5 | 4.2% | 3.3% |  | 0.9% | 3.8% |  |

Note: Level 1 (no problems), Level 2 (slight problem), Level 3 (moderate problem), Level 4 (severe problem), Level 5 (extreme problem).

Supplementary Figures A: Proportion of respondents reporting problems in each EQ-5D-5L dimensions by age group or by sex (only statistically significant p-values were noted)

1. Age 16-24

Between-wave:

p = 0.016 in Usual activity

Pooled vs population norms:

p < 0.001 in Self-care, Usual activities, and Anxiety/Depression

1. Age 25-44

Between-wave:

p = 0.005 in Anxiety/Depression

Pooled vs population norms:

p < 0.001 in all dimensions

1. Age 45-64

Between-wave:

p = 0.024 in Usual activity

Pooled vs population norms:

p < 0.001 in all dimensions except for Self-care

1. Age 65-74

Between-wave:

p = 0.025 in Mobility

p = 0.012 in Anxiety/Depression

Pooled vs population norms:

p < 0.001 in Mobility, Pain/Discomfort, and Anxiety/Depression

1. Age 75+

Between-wave:

p = 0.041 in Pain/Discomfort

Pooled vs population norms:

p = 0.023 in Usual activities

p < 0.001 in Anxiety/Depression

1. Men

Between-wave:

p = 0.020 in Usual activities

p = 0.035 in Anxiety/Depression

Pooled vs population norms:

p < 0.001 in all dimensions except Self-Care

1. Women

Between-wave:

p = 0.006 in Usual activities

p = 0.007 in Pain/Discomfort

Pooled vs population norms:

p < 0.001 in all dimensions

1. Urban

Sample vs population norms:

(Calgary) p < 0.001 in all dimensions

(Edmonton) p < 0.001 in all dimensions

1. Rural

Sample vs population norms:

p < 0.001 in all dimensions except the Self-care dimension

Supplementary Table 3. Weighted proportion of respondents (%) by EQ-5D-5L dimension and age

|  | Age group | | | | | |
| --- | --- | --- | --- | --- | --- | --- |
|  |  | 16-24  (Sample N = 455) | 25-44  (Sample N = 6411) | 45-64  (Sample N = 7596) | 65-74  (Sample N = 2833) | 75+  (Sample N = 751) |
| Mobility | Level 1 | 91.7% | 91.7% | 79.8% | 61.6% | 46.4% |
|  | Level 2 | 7.2% | 6.0% | 12.8% | 21.1% | 23.9% |
|  | Level 3 | 0.9% | 1.8% | 5.5% | 12.3% | 19.9% |
|  | Level 4 | 0.0% | 0.3% | 1.8% | 4.8% | 9.1% |
|  | Level 5 | 0.2% | 0.3% | 0.2% | 0.3% | 0.6% |
| Self-care | Level 1 | 87.3% | 93.0% | 93.5% | 91.6% | 86.4% |
|  | Level 2 | 11.0% | 5.1% | 4.6% | 5.7% | 8.6% |
|  | Level 3 | 1.4% | 1.6% | 1.5% | 2.2% | 3.4% |
|  | Level 4 | 0.2% | 0.3% | 0.2% | 0.3% | 1.0% |
|  | Level 5 | 0.2% | 0.0% | 0.1% | 0.2% | 0.6% |
| Usual activities | Level 1 | 47.9% | 59.5% | 65.9% | 61.6% | 52.4% |
|  | Level 2 | 25.2% | 22.1% | 19.8% | 23.7% | 27.0% |
|  | Level 3 | 19.0% | 13.3% | 9.8% | 10.5% | 15.9% |
|  | Level 4 | 4.6% | 3.3% | 2.7% | 3.0% | 3.3% |
|  | Level 5 | 3.3% | 1.8% | 1.8% | 1.1% | 1.4% |
| Pain/discomfort | Level 1 | 60.8% | 56.4% | 42.9% | 31.1% | 24.4% |
|  | Level 2 | 29.8% | 29.9% | 32.7% | 38.2% | 38.7% |
|  | Level 3 | 6.7% | 10.8% | 18.3% | 23.9% | 27.3% |
|  | Level 4 | 2.1% | 2.3% | 5.2% | 6.0% | 8.9% |
|  | Level 5 | 0.7% | 0.5% | 1.0% | 0.8% | 0.6% |
| Anxiety/depression | Level 1 | 16.9% | 24.2% | 33.4% | 47.2% | 55.7% |
|  | Level 2 | 27.7% | 36.0% | 39.4% | 36.2% | 29.4% |
|  | Level 3 | 31.7% | 27.9% | 20.0% | 13.6% | 12.2% |
|  | Level 4 | 14.6% | 7.7% | 5.1% | 2.3% | 1.9% |
|  | Level 5 | 9.1% | 4.3% | 2.1% | 0.7% | 0.7% |

Note: Level 1 (no problems), Level 2 (slight problem), Level 3 (moderate problem), Level 4 (severe problem), Level 5 (extreme problem).

Supplementary Table 4. Weighted proportion of respondents (%) by EQ-5D-5L dimension and sex or by EQ-5D-5L dimension and urban/rural residence

|  |  | Gender | | Urban/rural residence | |
| --- | --- | --- | --- | --- | --- |
|  |  | Man  (Sample N = 4652) | Woman  (Sample N = 13401) | Rural  (Sample N = 1544) | Urban  (Sample N = 6474) |
| Mobility | Level 1 | 84.1% | 80.3% | 80.7% | 82.3% |
|  | Level 2 | 9.8% | 12.0% | 13.1% | 11.0% |
|  | Level 3 | 4.4% | 5.5% | 4.9% | 4.8% |
|  | Level 4 | 1.4% | 2.0% | 1.2% | 1.6% |
|  | Level 5 | 0.3% | 0.2% | 0.1% | 0.3% |
| Self-care | Level 1 | 93.3% | 90.0% | 93.4% | 92.1% |
|  | Level 2 | 5.0% | 7.3% | 5.1% | 5.9% |
|  | Level 3 | 1.4% | 2.1% | 1.3% | 1.7% |
|  | Level 4 | 0.2% | 0.4% | 0.1% | 0.3% |
|  | Level 5 | 0.1% | 0.2% | 0.1% | 0.1% |
| Usual activities | Level 1 | 62.7% | 56.1% | 65.3% | 58.7% |
|  | Level 2 | 20.4% | 24.3% | 19.4% | 23.0% |
|  | Level 3 | 12.3% | 13.6% | 13.2% | 13.8% |
|  | Level 4 | 2.5% | 4.1% | 1.7% | 3.2% |
|  | Level 5 | 2.0% | 1.8% | 0.4% | 1.3% |
| Pain/discomfort | Level 1 | 52.6% | 44.1% | 48.4% | 49.1% |
|  | Level 2 | 31.3% | 32.9% | 30.5% | 33.1% |
|  | Level 3 | 12.4% | 17.3% | 16.5% | 13.2% |
|  | Level 4 | 3.3% | 4.6% | 4.1% | 3.8% |
|  | Level 5 | 0.4% | 1.1% | 0.5% | 0.8% |
| Anxiety/depression | Level 1 | 35.3% | 24.8% | 37.7% | 27.5% |
|  | Level 2 | 33.6% | 37.2% | 30.2% | 35.0% |
|  | Level 3 | 21.0% | 26.5% | 23.4% | 25.2% |
|  | Level 4 | 6.1% | 8.0% | 6.1% | 8.6% |
|  | Level 5 | 4.0% | 3.5% | 2.6% | 3.8% |

Note: Level 1 (no problems), Level 2 (slight problem), Level 3 (moderate problem), Level 4 (severe problem), Level 5 (extreme problem).

References

1. Statistics Canada. (2017, November 29). Census Profile. 2016 Census. Statistics Canada Catalogue no. 98-316-X2016001. Retrieved from https://www12.statcan.gc.ca/census-recensement/2016/dp-pd/prof/index.cfm?Lang=E

2. Government of Alberta. (2017). *2016 Census of Canada - Population and Dwelling Release*. Retrieved from https://open.alberta.ca/dataset/7d02c106-a55a-4f88-8253-4b4c81168e9f/resource/e435dd59-2dbd-4bf2-b5b6-3173d9bd6c39/download/2016-census-population-and-dwelling-counts.pdf

3. Government of Alberta. (2020). Unemployment Rate. Economic Dashboard. Retrieved from https://economicdashboard.alberta.ca/unemployment

4. Government of Alberta. (2020). *COVID-19 Alberta statistics*. Retrieved from https://www.alberta.ca/stats/covid-19-alberta-statistics.htm#total-cases

5. Government of Alberta. (2021). Interactive Health Data Application. Retrieved from http://www.ahw.gov.ab.ca/IHDA_Retrieval/selectSubCategory.do

6. Statistics Canada. (2021, September 8). Table 13-10-0096-01 Health characteristics, annual estimates. Retrieved from https://doi.org/10.25318/1310009601-eng

7. Statistics Canada. (2021, April 21). Table 13-10-0383-01 Prevalence of cannabis use in the past three months, self-reported. Retrieved from https://doi.org/10.25318/1310038301-eng

8. Alberta PROMs & EQ-5D Research & Support Unit (APERSU). (2018). *Alberta Population Norms for EQ-5D-5L*. Edmonton, Canada: School of Public Health, University of Alberta. Retrieved from https://apersu.ca/wp-content/uploads/2021/02/Alberta-Norms-Report_APERSU.pdf
